# Periodicity, Elliott waves, and fractals in the NFT market

**DOI:** 10.1038/s41598-024-55011-x

**Published:** 2024-02-23

**Authors:** J. Christopher Westland

**Affiliations:** University of Illinois Chicago, Chicago, USA

**Keywords:** Engineering, Mathematics and computing

## Abstract

Non-fungible tokens (NFTs) are unique digital assets that exist on a blockchain and have provided new revenue streams for creators. This research investigates NFT market inefficiencies to identify claimed cyclic behavior and cryptocurrency influences on NFT prices. The research found that while linear models are not useful in modeling NFT price series, models that extract periodic behavior can provide explanations and predictions of price behavior. The investigation of autocycles in cryptocurrency and NFT markets did not support the existence of Elliott Wave behavior in any of these blockchain enabled assets. Rather NFT price behavior is strongly tied to the underlying asset and its community of fans. These fans commit to periodic bouts of idiosyncratic trading which cools for a while, and then restarts. The research found no evidence supporting whole market effects across the full price series of individual NFTs. The research strongly supports prior findings that the offsetting movements significantly influence NFT prices and trading volume in Bitcoin and Ether. The research found NFT markets exhibit characteristics resembling a social media platform rather than more traditional asset markets like stock exchanges. It found that traditional linear econometric models cannot predict or explain NFT price series, only that NFT price and volume were weakly correlated. Fractal models consistent with Elliott wave theory do explain some of NFT price behavior, but are not consistent or stable over time. This research confirmed prior research findings that Bitcoin and Ether price movements are correlated with general NFT price and volume series in periods of between 24 and 48 h, with significant numbers of trades into and out of cryptocurrencies at 2 and 8 h.

## Introduction

Non-fungible tokens (NFTs) are unique blockchain assets that offer new revenue streams for creators at the expense of a potentially large environmental footprint, and potential risks from poor security and market regulation. NFTs’ distributed ledger allows for allegedly secure and transparent record-keeping which NFT proponents argue are safe, tamper-proof, and cheaply transferable. Recent thefts and hacks have brought additional scrutiny to such claims.

“Studies of the nonfungible token (NFT) market have investigated price behavior using a variety of analytical methods. Positive but sometimes contradictory causal behaviors have been found to influence NFT prices in^1–10^, while economic models in^6,11–14^ and^15^ suggest there is little correlation between NFTs and real world asset markets, including those for physical painting, sculpture and objet d’art.

In other asset markets, similar pricing models favor analysis only of the price series, i.e., “technical” analysis^16^, over causal impact of real world events, i.e., “fundamental” analysis^17^. Elliott wave technical analysis is an active area of quantitative research in collective trader psychology, that tracks market optimism and pessimism in repeating sequences of intensity and duration. Elliott proposed that mood swings create patterns in the price movements of markets at every degree of trend or time scale.

Numerous recent studies have concluded that it may be possible to predict prices with Elliott wave technical analysis in diverse markets, including^18–41^ and^42^. The methodology in these research papers has unfortunately been limited by a dearth of modern quantitative models to completely describe the Elliott waves. Elliott recommended^18^ Fibonacci series in the 1930s, and they are commonly employed for analysis today. But today we also have rich quantitative models that can more completely capture the Elliot wave series such as fractal analysis and wavelet models using software tools that have only become available over the past 20 years.

The current research studies the existence of Elliott wave like behavior in NFT markets, where I employ wavelet and fractal models to represent Elliott wave models of technical price movements in OpenSea’s NFT market. The research looks at Elliott wave like price autocycles, as well as price cycle correlations between NFTs and cryptocurrencies which have been suggested in the conclusions of prior studies. Elliott, Mandelbrot and Taleb all recognized this, and sought out the extra dimensions of prices through recursive “fractal” theories of asset markets^43–47^. Furthermore, their models of human behavior are embraced by many whose living depends on understanding these models.

This study builds on the results of recent research in both explaining and predicting nonfungible token (NFT) pricing and returns, and addresses some of the open questions applying an approach which has been successful in forecasting other financial asset markets. The research is structured to answer six research questions concerning NFT markets:

- Do linear econometric models accurately predict the price behavior in NFT markets, e.g., $ARIMA\left( {p,d,q} \right)$ time series models, or the capital asset pricing model^48^? The capital asset pricing model^48^ (CAPM) is an old model in finance that has had enormous influence over its existence, and which is used to determine a theoretically appropriate required rate of return of an asset, to make decisions about adding assets to a well-diversified portfolio. The model takes into account the asset’s sensitivity to non-diversifiable risk and the expected return of the market and the expected return of a theoretical risk-free asset. (Linear models provide my baseline model for NFT price behavior)
- Does Elliott wave like price behavior exist in NFT markets?
- Do fractal representations of Elliott wave like behavior capture NFT autocyclic price behavior? (Elliott wave model tests)
- Do wavelet representations of Elliott wave like behavior capture NFT autocyclic price behavior? (Elliott wave model tests)
- Are there time-varying correlations (i.e., wavelet coherency) between Bitcoin and Ether cryptocurrency market price movements and NFT price and volume movements, e.g., as suggested by Ante^49^ and others. If so, over what time-periods? (Tests of Ante’s findings)
- Is NFT price correlated with trading volume (General test of a common asset market correlation).

NFT markets have been found to suffer from significant inefficiencies^50–52^. This research investigates this inefficient market to identify speculated cyclic behavior and cryptocurrency influences on NFT prices. The current research proceeds as follows. Section “Background” of this paper surveys prior research and provides background for the research questions. Section “Data and methodology” describes the dataset and methodology. Section “Results” presents the result of analysis on all of the research questions. Section “Findings and conclusions” summarizes the conclusions of analysis, and section “Future research” suggests future avenues of research and how they would be useful both for practitioners, regulators and traders, as well as expanding the academic field of digital markets.

## Background

The concept of an NFT has been around since the early days of blockchain technology and has recently seen significant trading volume in art and collectibles. In 2012, NFTs known as *Colored Coins* were created, followed in 2014 by the *Counterparty* platform and the emergence in 2017 of digital marketplaces for creators, such as *CryptoPunk* and *CryptoKitties.* CryptoKitties was covered by CNN, MSNBC, and The New York Times, and inspired the creation of the *OpenSea* trading platform. NFTs were relatively inexpensive until the 2021 announcement and sale of Beeple’s “Everydays” NFT which garnered the third-highest auction price ever earned by a living artist. NFT sales reached $18 billion by the end of 2021. This represented a significant increase from the previous year, when total NFT sales were less than $2 billion.

The rapid expansion of investments into non-fungible tokens (NFTs) over the past five years has motivated a substantial body of research into price setting behavior, influences of other markets on NFT prices, and social and networking influences on NFT prices. Economic models of NFT markets have been developed and explored in^6,11^ and^12^ who found support for the nonfungibility of NFTs. Found low to nonexistent correlation between NFTs and cryptocurrencies^13^ and^14^. Yousaf et al.^15^ explored the correlation of metals trading and NFT returns.

Behavioral, social and information networking influences on market prices have a long history of anecdotal support, but rigorous analysis has been elusive due to the subjectivity of data. There is an evolving stream of academic research:^1^ investigated ‘herding’ behaviors in markets such as *Cryptokitties* and *Godsunchained* assets, finding specific patterns of herding among investors;^2^ looked at spatial heterogeneity in price setting, supporting the idea that NFTs are truly non-fungible;^3^ studies centrality measures, clustering coefficient, and assortativity in NFT markets, showing that these were highly unstable over time and moved by relatively few investors, a result suggestive of active market manipulation, where^4^ and^5^ also provide strong arguments for this interpretation;^6^ provides evidence that pricing and returns are strongly influenced by pump-and-dump schemes;^7^ found that either economic fundamentals or investor attention can increase the volatility of NFTs significantly;^8^ explored the behavioral time-varying and cross-section properties of NFT market liquidity;^9^ found no correlation between NFTs and the physical art market, while^10^ found that the counter-cyclical pricing of NFTs made it a hedge against other assets in a portfolio.

Literature reviews appear in^14,53–56^ for general literature reviews. With regard to intellectual property protection^57^. Provides histories^58^. Surveys NFTs in healthcare, with suggestions for future applications^59^ and^60^. Beyond this, there is a substantial strain of semi-speculative academic literature that questions whether NFTs are an asset at all, or merely a mathematical oddity and perhaps the most recent fad in market profiteering^61–68^. In a related stream, NFT in branding and advertising has been studied in^69^.

Ante^49^ examined cryptocurrency market movements and NFT price and volume, finding that NFT prices and trading volume were significantly influenced by external factors such as the price and volume movements of Bitcoin and Ether, as well as overall market sentiment. Specific sources of inefficiencies were observed in Pereira et al.^52^. Ante’s research asserted that when Bitcoin and Ether experienced large price and volume movements, NFT prices and trading volume also tended to rise or fall. Additionally, the study found that NFT prices were significantly higher when the market sentiment was positive, as measured by the *Crypto Fear and Greed Index.* The study also found evidence of risk management behavior by NFT traders, as they tended to sell NFTs and move into Bitcoin or Ether during periods of high volatility or uncertainty in the cryptocurrency markets, suggesting that NFT traders might use Bitcoin and Ether as a hedge against market risk. Ante’s^49^ dataset comprised only 1231 daily observations (January 1, 2018–May 16, 2021) on the volume of NFT sales in USD, the number of blockchain wallets holding or interacting with NFTs on a particular day, and the prices of Ether (ETH) and Bitcoin (BTC) in USD. Ante’s study’s daily resolution gave excessive weight to open and close prices set by the market, and was limited to a period in which NFTs were less prominent prior to May 2021. Ante’s^49^ data had a minimum daily resolution, whereas efficiencies in markets like OpenSea have made intraday (minute-by-minute) trading more important for tracking trends and influences. Nonetheless, NFT markets are highly inefficient^52^ and idiosyncratic, having more in common with social media than liquid markets. The current research attempts to resolve these shortcomings by analyzing a curated database of 6,071,027 min-by-minute NFT transactions through the end of 2022, along with contemporaneous Bitcoin and Ether minute-by-minute price and volume datasets.

Price cycle analysis in asset markets relies on several ad hoc technical analysis methods in practice. The most popular and prominent of these is Elliott Wave theory, which uses a fractal-like method for predicting the length and frequency of ‘bull’ and ‘bear’ market swings^18^. Elliott Wave theory studies patterns of changes in investor sentiment and psychology, which claims that investor sentiment cycles in predictable, recurring fractal patterns (“waves”) in financial markets^43^. Elliot Wave theory is influential to this day in finance, most recently popularized in^44^. Several large financial funds trade on Elliott wave theory^45–47^. Fractal dynamics of asset prices create a time and frequency resolution dilemma, resulting from the Heisenberg uncertainty principle, and wavelets offer the most comprehensive formal mathematical approach for analyzing dynamics in such domains^70–74^. Wavelets are used in the empirical analysis of fractals datasets to measure local characteristics. In this research I take advantage of wavelets methods that allow perusing the local structure of a fractal^75–79^. This ability is important because asset markets, such as stock and Forex markets exhibit fractal behavior^70–72^. It is additionally important that the Morlet wavelets applied here are closely related to human perception, hearing and vision, which is consistent with Elliott waves having their basis in human perception^80,81^. The wide acceptance of fractal and Elliott wave models of asset market price movements suggest that fractal and wavelet empirical analyses of NFT markets can provide insights into the detailed nature of inefficiencies. Wavelets provide a formal mathematical model for analyzing fractal-like price swings in asset markets.

## Data and methodology

The current research investigates price and trading volume movements in the NFT market, and possible external influences on price and volume from whole market effects and risk management through open market trade of Bitcoin and Ether whose Ethereum blockchain accounts for the majority of NFT trades. The research uses a curated database of 6,071,027 NFT transactions of 4,337,718 unique NFTs on OpenSea, the world’s largest NFT marketplace, between 6:44 pm UTC on November 11, 2017 and midnight on April 28, 2021^82^. OpenSea is an online peer-to-peer marketplace that went live in New York on December 20, 2017. Additionally, the research collected contemporaneous Bitcoin and Ether price and volume datasets.

**Figure 1:**
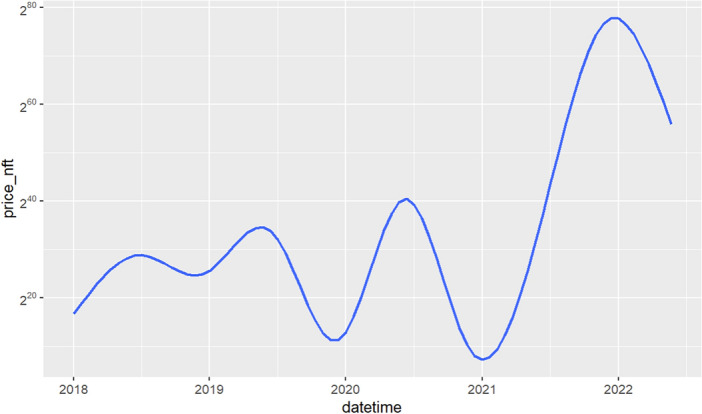
Smoothed average NFT prices for the whole market.

**Figure 2:**
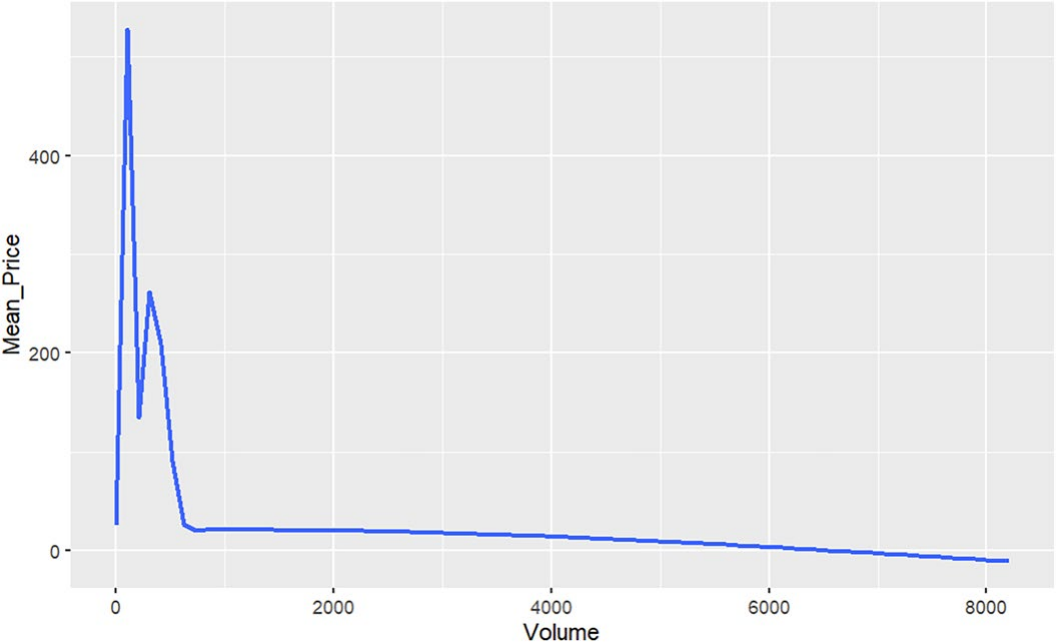
Volume vs. mean price for All NFTs.

**Figure 3:**
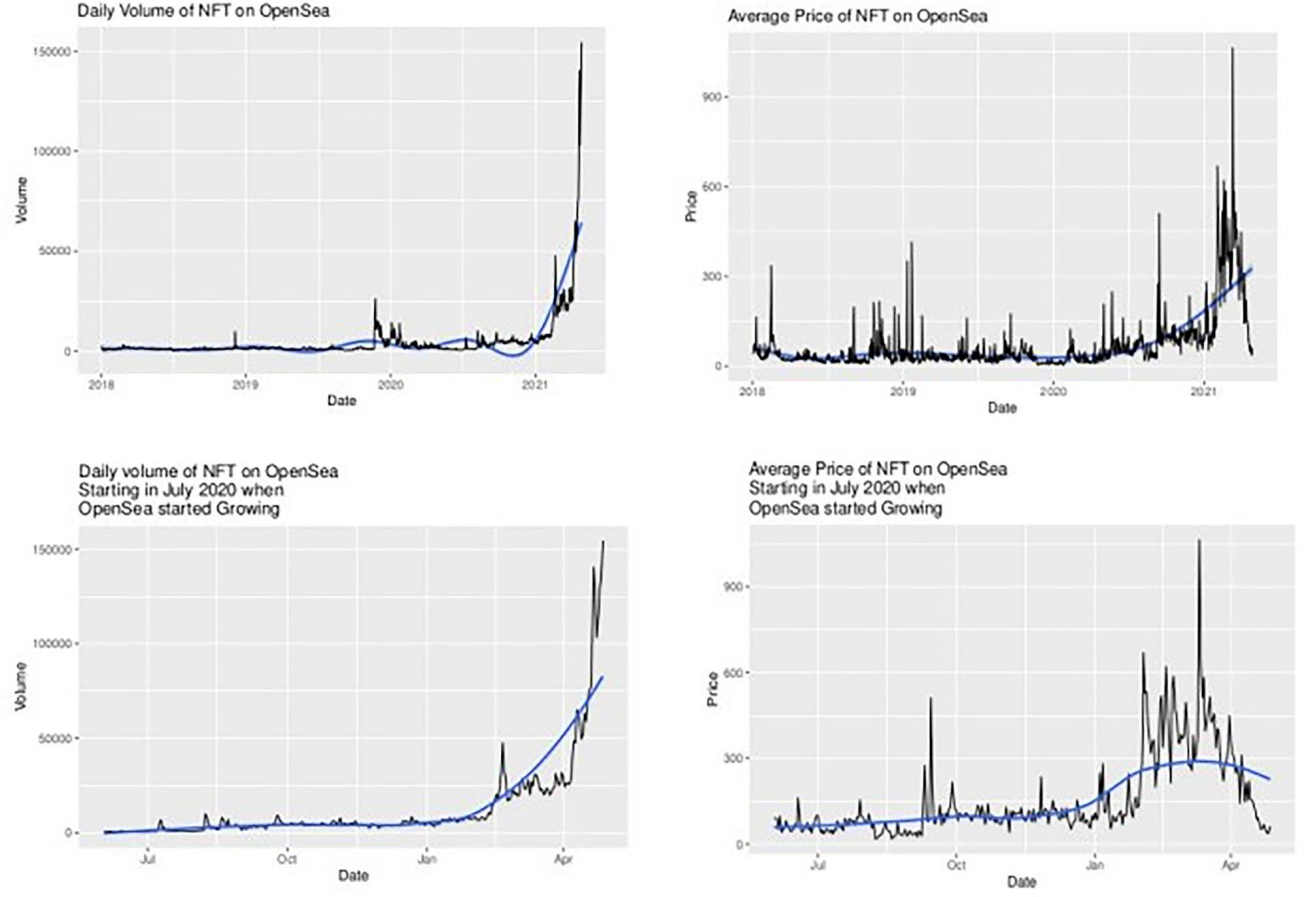
Price and volume graphs for NFT dataset (2018–2022 top; 2020–2022 bottom).

**Figure 4:**
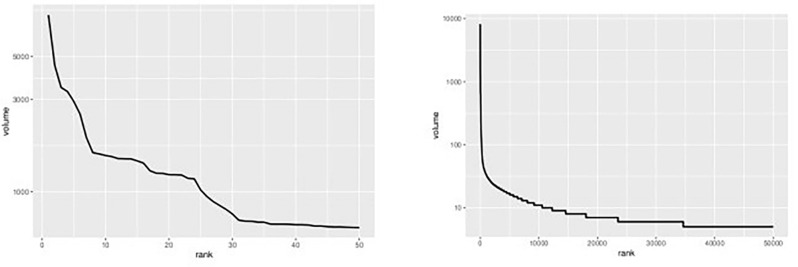
Rank ordered total volume of trading for first 50 (left) and first 50,000 (right).

**Figure 5:**
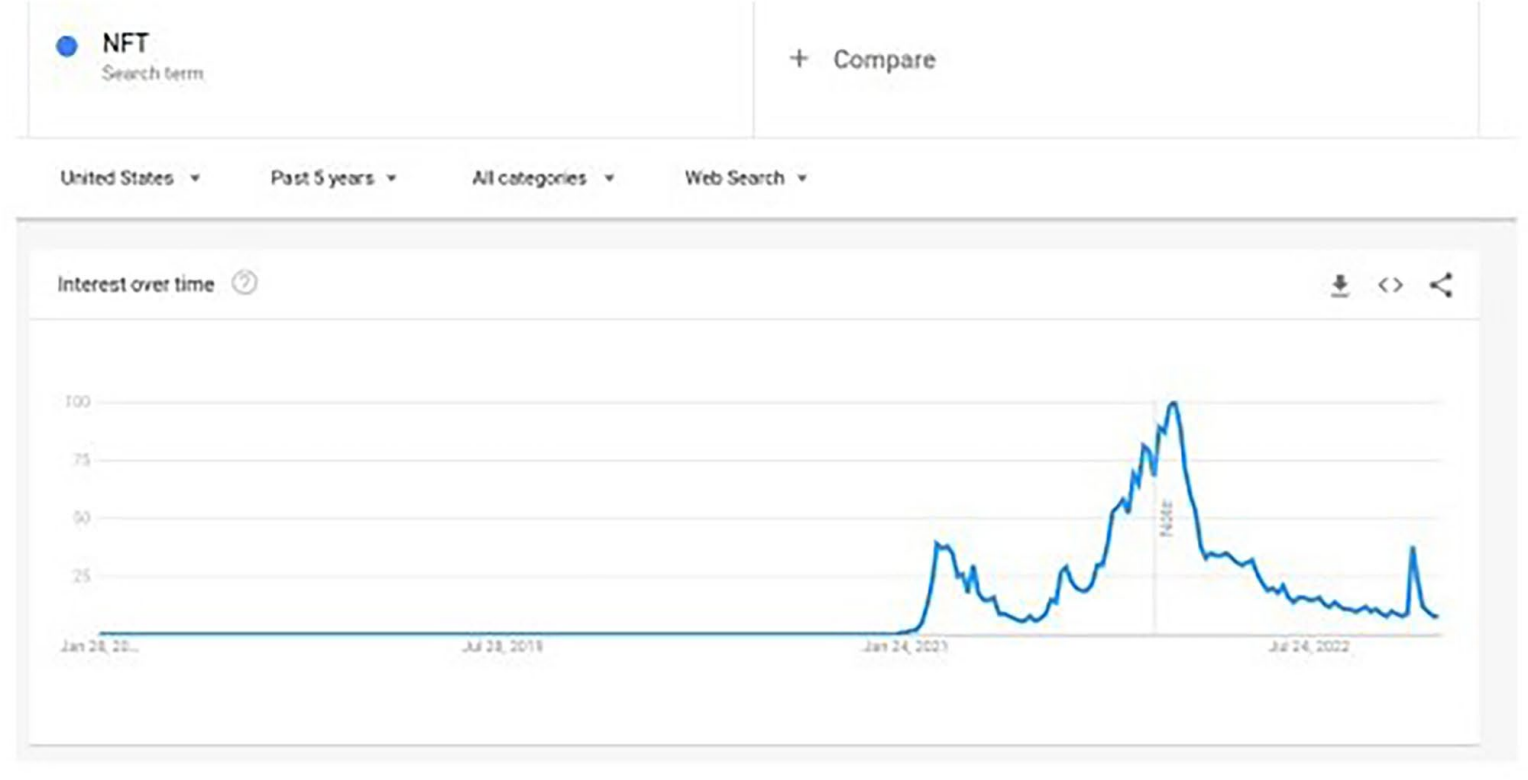
Google trends for NFTs. Google Trends is a website by Google that analyzes the popularity of top Google search queries using graphs to compare the search volume of different queries over time.

Figure 1 displays, on a log-linear graph, the smoothed prices of NFTs over time. Figure 2 displays volume-price statistics showing that only a few hundred of the 4.3 million NFTs are relevant to the market; indeed, many NFTs never trade, and 82% of NFTs see only 1 trade over their lifetime; 99% see less than 5 trades. Only a few NFTs are ever of any value, and the market and its importance are idiosyncratic and less important than the popular press has conveyed. Figure 3 displays four graphs of NFT price and volume data over time, emphasizing in particular periods of high activity. Figure 3’s top two panels show volume and price data for he full time of OpenSea’s operation, while the bottom line restricts these to the elevated price period starting around the time of Beeple’s *Everydays.* Figure 4 rank orders the volume of trading for the top 50 NFTs (left) and the top 50,000 NFTs (right), showing that only a small number of NFTs drive the entire market. Given the high dimensionality of this data, graphic reporting of statistics is most direct, and tables of the same data would be messy and voluminous, thus are minimized in the current research. Note that prices and volumes follow trends in Google search shown in Fig. 5.

The analyses performed in this research require relatively large datasets of prices to produce reliable results. Figure 4 shows that such transaction volumes exist only for the top ranked NFTs. In addition, unlike stock exchanges and other liquid markets, NFTs are faddish items, and only a few of these fads actually gain traction. Market wide effects are minimal, and individual NFTs demonstrate unique trading behaviors. To address these unique aspects of the NFT market, the current research analyzes activity in only the top 10 ranked NFTs, in order to have large enough datasets to provide reliable estimates, and as a way to investigate the unique behaviors of each individual NFT market. Tables 1 and 2 provide details on the NFTs used in the analysis: table one provides a link to the OpenSea webpage that contains details and pictures where applicable, of the 10 most traded NFTs; Table 2 displays trading statistics for those 10 NFTs. Prices have generally increased over time.

**Table 1:** URLs of the top 10 NFTs ranked by trading volume.

Rank	URL of non-fungible token OpenSea description page
1	https://opensea.io/assets/0xbb5ed1edeb5149af3ab43ea9c7a6963b3c1374f7/0
2	https://opensea.io/assets/0x5d0ee539893323f9965e3ac2846ea6421255ae79/0
3	https://opensea.io/assets/0x079b18d6556e198c98e599490c6cbba231ab4db8/0
4	https://opensea.io/assets/0xcd65bacacea37e65eac019f37f5cb38ae62a7fbf/0
5	https://opensea.io/assets/0xcd65bacacea37e65eac019f37f5cb38ae62a7fbf/0
6	https://opensea.io/assets/0xcd65bacacea37e65eac019f37f5cb38ae62a7fbf/0
7	https://opensea.io/assets/0x079b18d6556e198c98e599490c6cbba231ab4db8/0
8	https://opensea.io/assets/0xcd65bacacea37e65eac019f37f5cb38ae62a7fbf/0
9	https://opensea.io/assets/0xcd65bacacea37e65eac019f37f5cb38ae62a7fbf/0
10	https://opensea.io/assets/0xcd65bacacea37e65eac019f37f5cb38ae62a7fbf/0

**Table 2:** Trade Statistics for the Top 10 NFTs ranked by trading volume.

Rank	First trade	Last trade	Volume	Low price	High price
1	2018-09-11 11:05:00	2018-12-08 05:33:00	32,122	0	2.663e+26
2	2019-07-11 07:00:00	2019-07-16 23:30:00	12,688	0	3.492e+25
3	2018-07-10 01:16:00	2018-07-17 15:00:00	10,904	0.2343	9.185e+ 19
4	2018-08-07 07:00:00	2018-08-26 06:59:00	8742	0	1.627e+21
5	2017-10-25 19:08:00	2021-04-27 07:00:00	8206	0	1.735e+27
6	2018-02-16 13:44:00	2018-07-10 01:16:00	7521	0	1.077e+27
7	2018-08-26 06:59:00	2018-09-07 05:11:00	7039	0	2.69e+20
8	2018-02-05 07:01:00	2018-02-16 13:44:00	6911	0	3.017e+20
9	2018-09-07 05:11:00	2018-09-11 11:05:00	6115	0	5070
10	2017-06-23 21:05:00	2017-10-25 19:07:00	5862	0	2.243e+21

**Figure 6:**
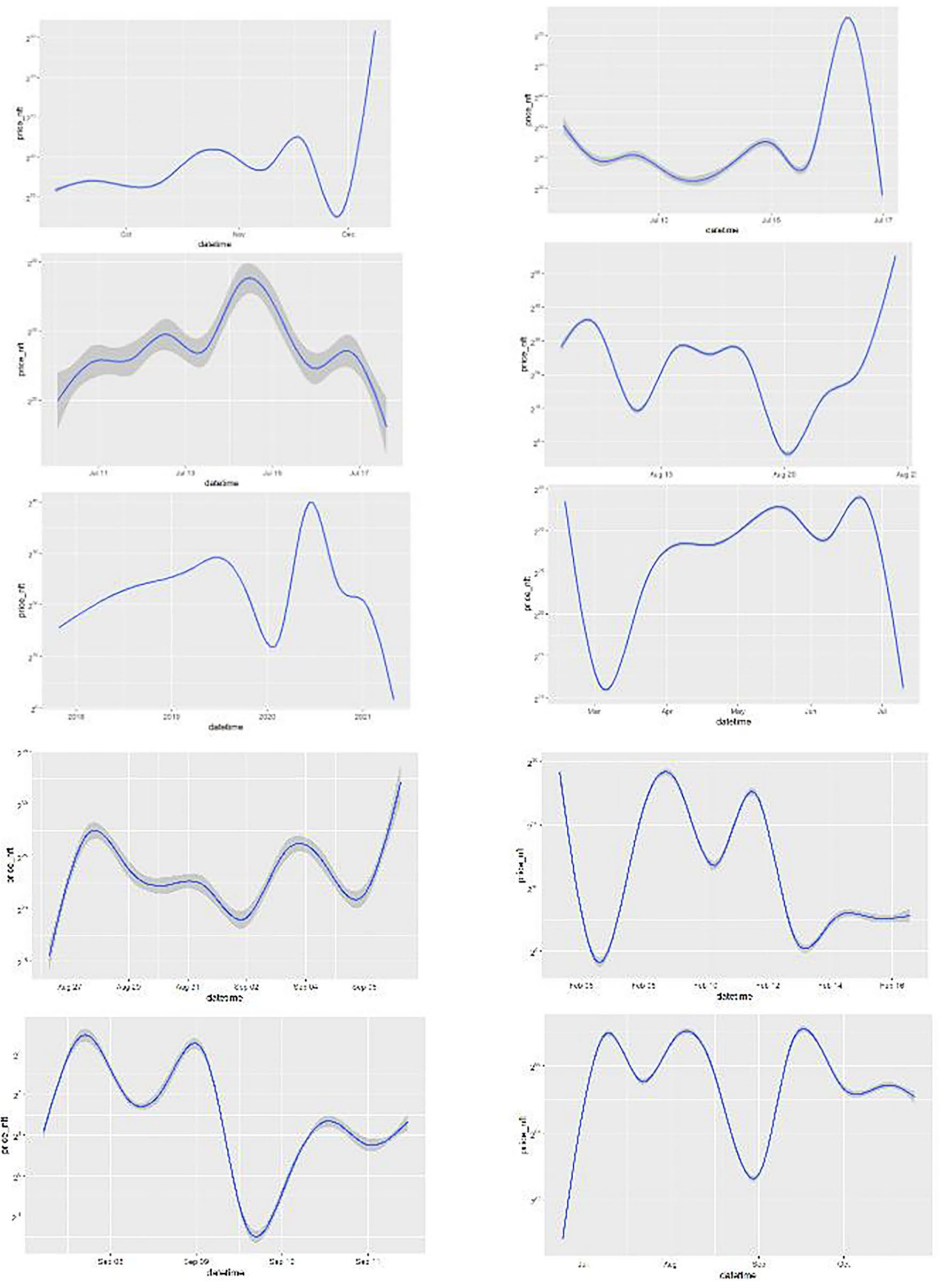
Moving average of top 10 NFT prices during their trading period.

Figure 6 indicates of how NFTs differ, by showing smoothed price series over their trading lives. The panels (an all of those of other grid figures) show each of the top 10 NFT price streams in rank order:

$$\left[ {\begin{array}{*{20}c} {rank\;1} & {rank\;2} \\ {rank\;3} & {rank\;4} \\ {rank\;5} & {rank\;6} \\ {rank\;7} & {rank\;8} \\ {rank\;9} & {rank\;10} \\ \end{array} } \right]$$

**Figure 7:**
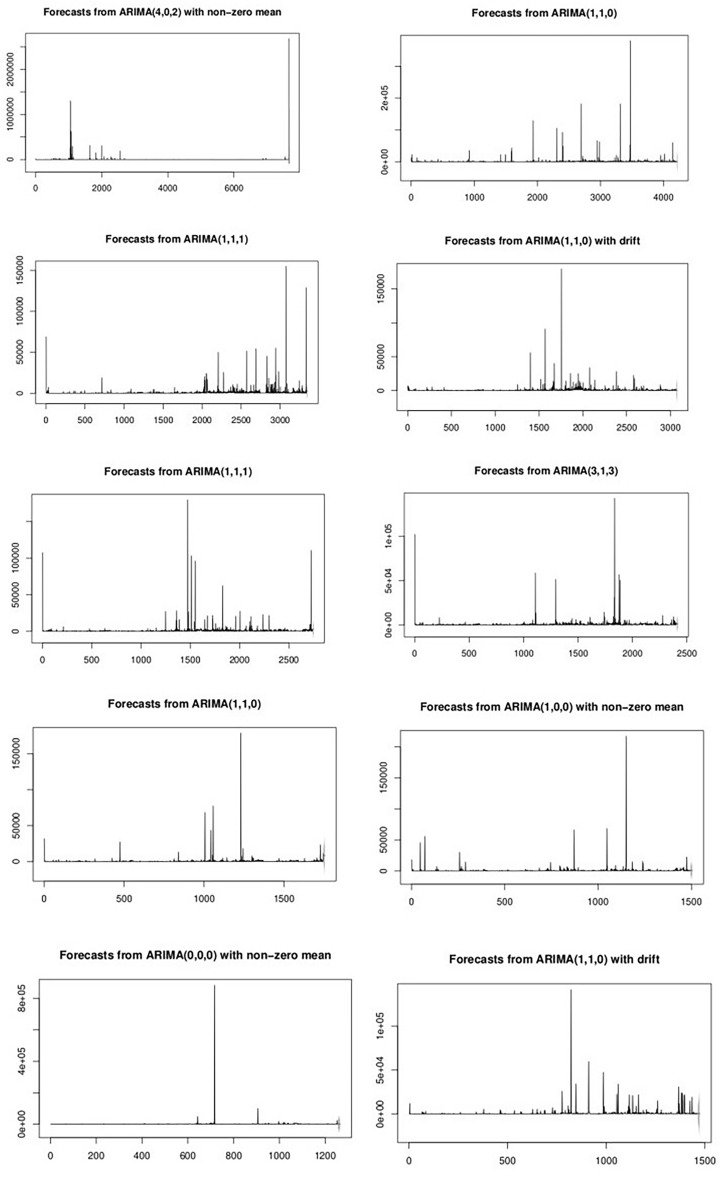
Optimized ARIMA Price Forecasts for the Top 10 NFTs.

**Figure 8:**
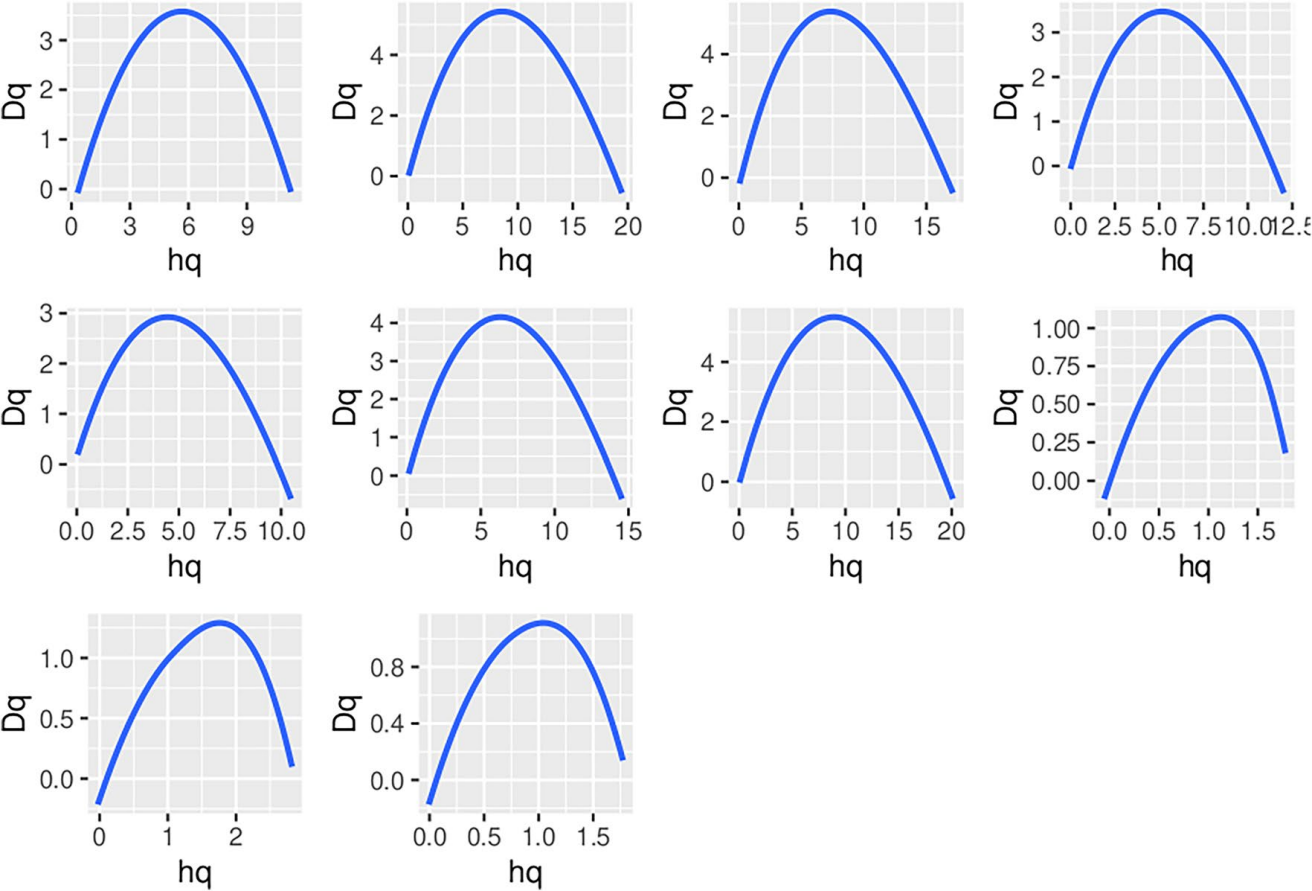
Multifractal Singularity Spectra for the Top 10 NFTs, where behavior around any point is described by a local power law ${\text{s}}\left( {{\text{q}} + {\text{a}}} \right) - {\text{s}}\left( {\text{q}} \right) \sim {\text{a}}^{{{\text{h}}\left( {\text{q}} \right)}}$ and which fully describes the statistical distributions of the price series. The x-axis ${\text{h}}_{{\text{q}}}$ describes the local degree of singularity or regularity around the point ${\text{q}}$. The y-axis ${\text{D}}_{{\text{q}}}$ displays the fractal dimension at ${\text{q}}$.

**Figure 9:**
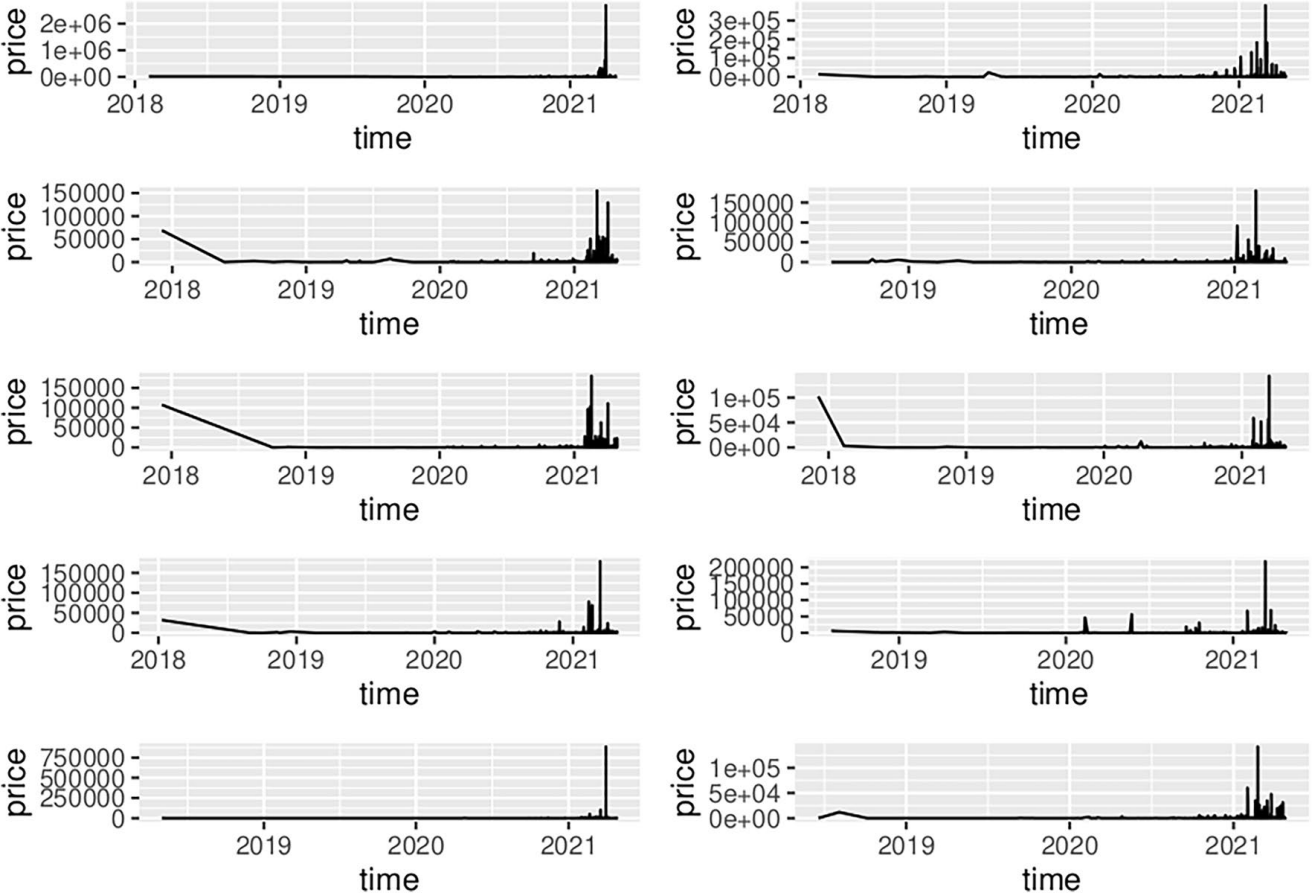
Price levels over the time period of the dataset used in this research.

**Figure 10:**
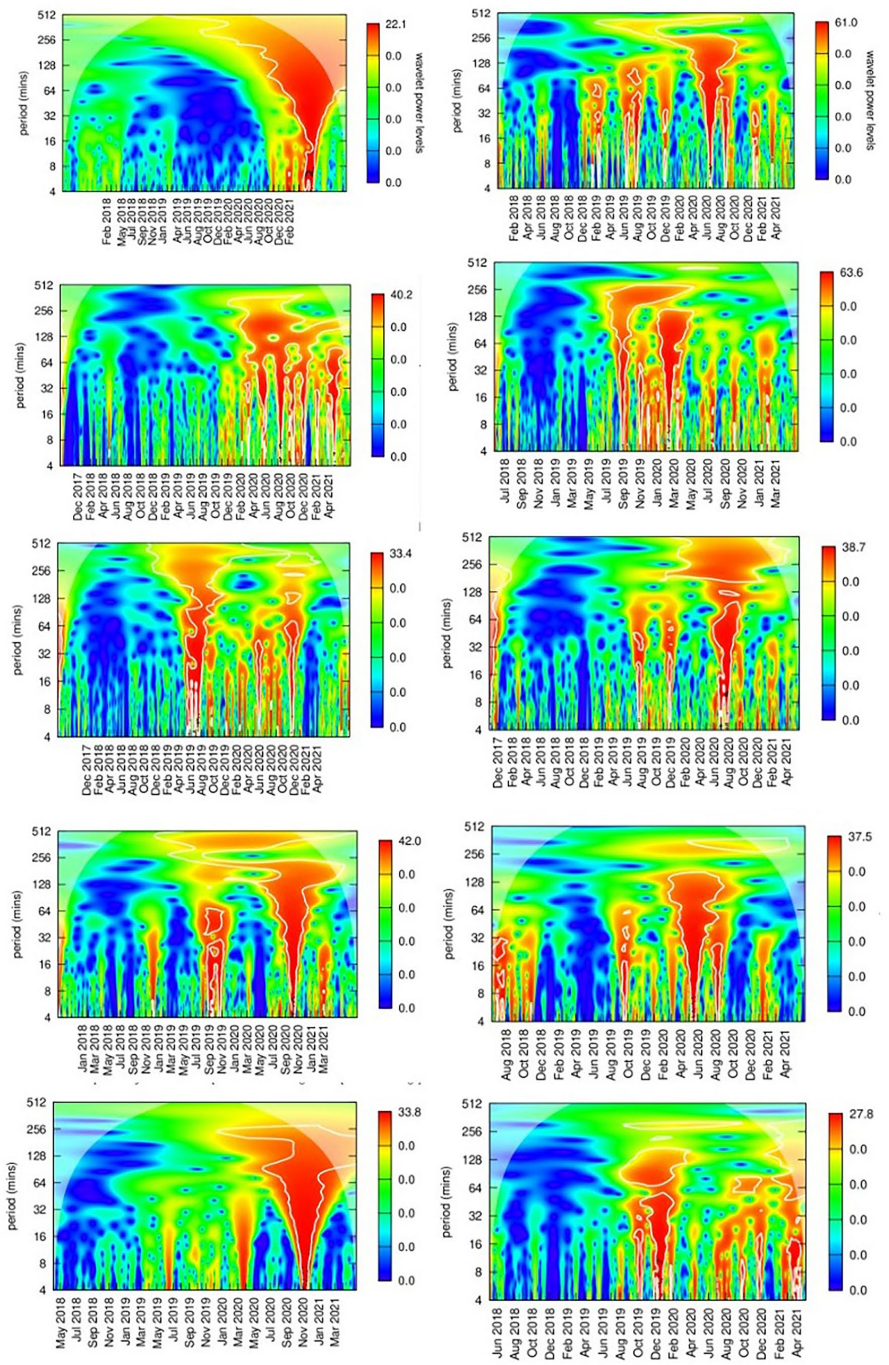
Wavelets Autocycle Power Graphs for the Top 10 NFTs Between 4 and 512 min.

**Figure 11:**
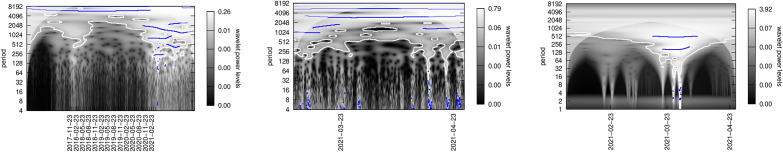
Price autocycles for ether, bitcoin and whole NFT market respectively.

**Figure 12:**
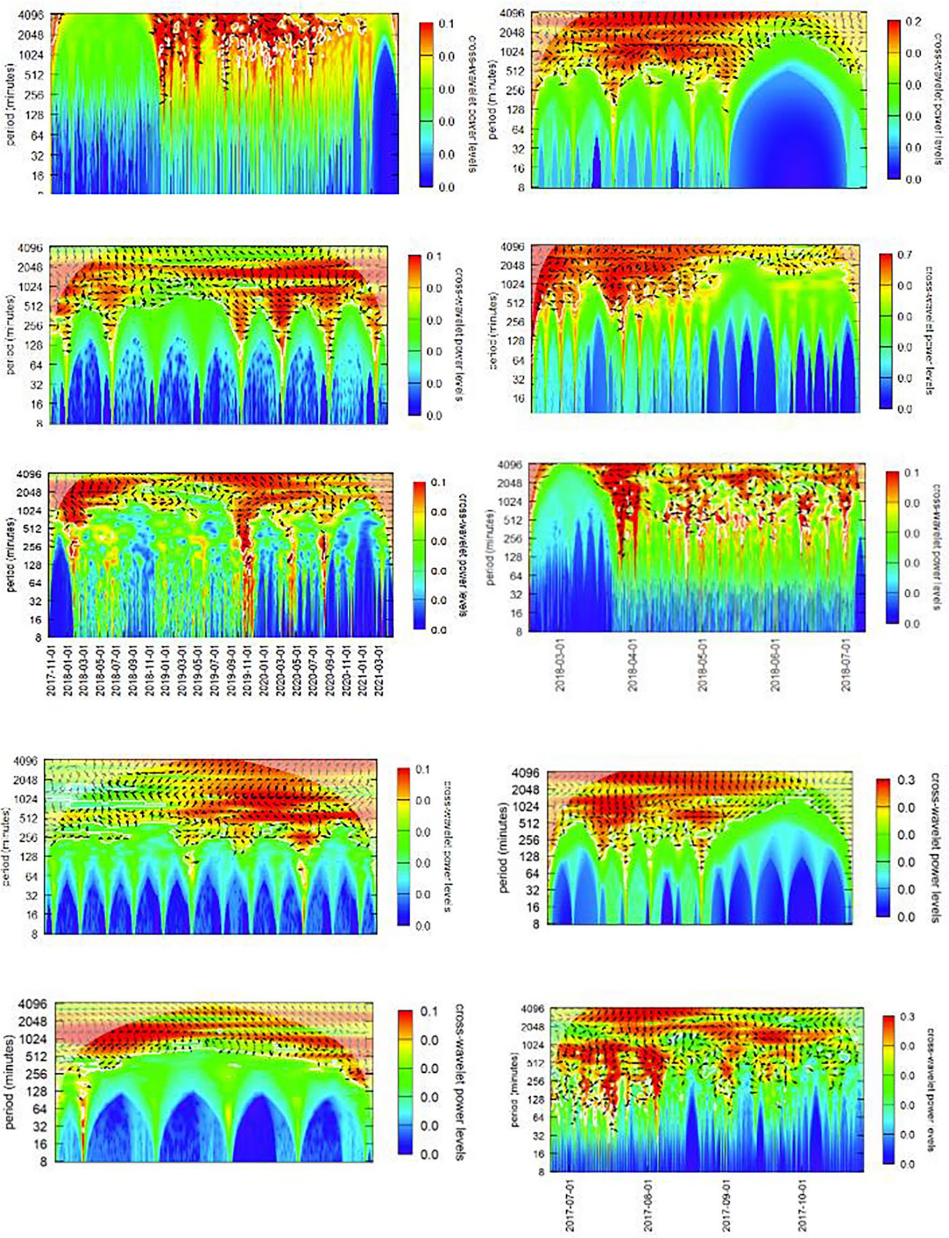
Ether price coherency (correlation) with the Top 10 ranked ranked NFTs.

Note that the time periods represented in each of the panels are different; the reader should refer to Table 2 to calibrate the reading of timelines in the grid panels in Figs. 6, 7, 8, 9, 10, 11 and 12 which all share the sequence shown in the grid matrix above.

**Figure 13:**
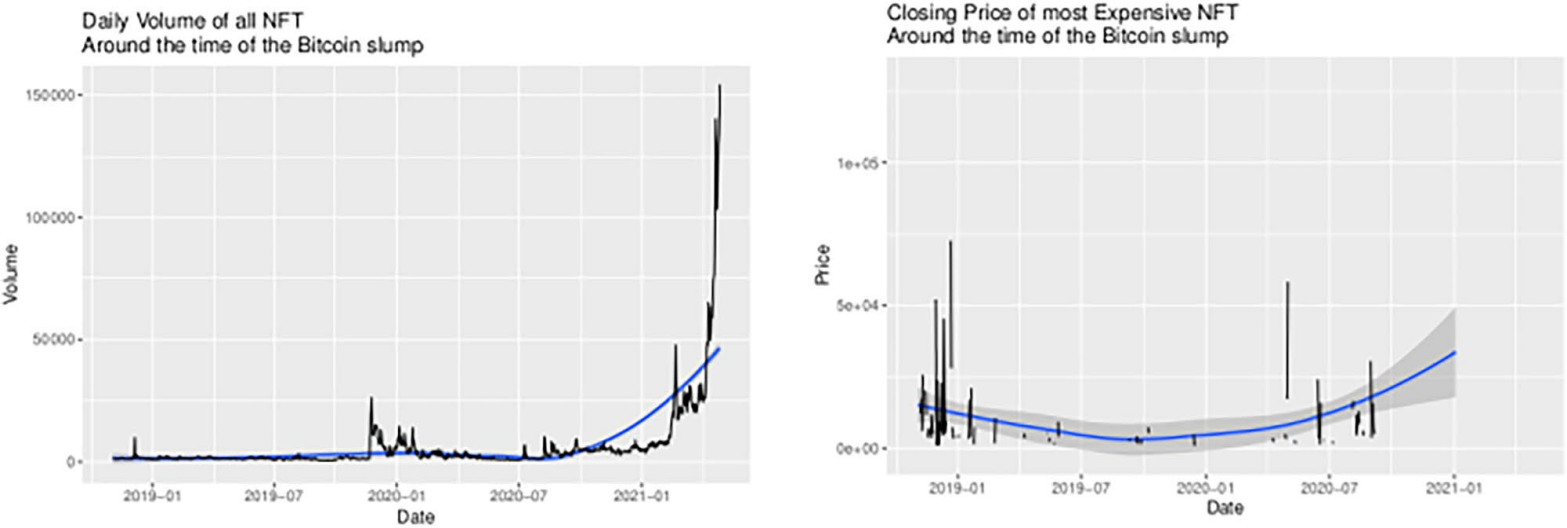
NFT trading around the time of the bitcoin slump.

In the exploratory charts in Fig. 13 there is a single unusual event-specific price spike that seems to roughly correspond to the 2019 crash in the cryptocurrency market. In 2018, hackers successfully attacked Japan’s largest cryptocurrency market *Coincheck*, stealing $530 million. Subsequently, *Coincheck* indefinitely suspend trading. In the same year, cryptocurrency markets suffered a wide range of abuses causing investor losses. A negative Ernst & Young market study further fueled skepticism of blockchain assets, suggesting that criminal activity was stealing $1.5 million per month in ICO proceeds, or $400 million in capital. This fomented a general sell-off of cryptocurrencies, with a possible transfer of value into NFT markets. From around $20,000 level, the sell-off drove Bitcoin to plunge as low as around $3,000 in December 2018, and had been dubbed the great crypto crash of 2018, and this overall negative climate lasted through 2019. In addition, Bitcoin “halving” was thought to play a role in this crash. Halving limits Bitcoin supply, pushing up demand and prices as investors enter the market. There is typically an uptrend and peak in Bitcoin—and subsequently in other cryptocurrencies—after a halving event which happens around every 4 years. Since Bitcoin’s inception in 2009, the halving events every four years in 2012, 2016 and 2020 result in Bitcoin price hikes months to a year after it happened. This is followed by a slump, two years after the halving event. This slump occurred very quickly in 2018 and possibly influenced a restructuring of cryptocurrency portfolios by investing more in NFTs. Since the NFT market was relatively small in 2018, this could have represented a significant percentage of transaction volume for the NFT market.

## Results

### Baseline linear time-series models

As a baseline comparison for wavelets and fractal analysis, the research analyzed the price time series for each of the 10 most popular NFTs using ARIMA models to assess the method which extracts the greatest amount of information. If Elliott Wave or fractal theories of price series are valid, then they should outperform our baseline series.

I denote the observed NFT price series by $p_{1} ,p_{2} ,...,p_{n}$. A forecast of $p_{t + h}$ based on all of the data up to time $t$ is denoted by $\hat{p}_{t + h|t}$. Figure 7 graphically shows ARIMA forecasts of each of the top 10 NFT price streams over the span of the dataset and Table 3 shows fit statistics. For each price stream, the *auto.arima* algorithm in *R*’s *forecast* package computed the optimal $ARIMA\left( {p,d,q} \right)$ model, where $p$ is the order (number of time lags) of the autoregressive model, $d$ is the degree of differencing (the number of times the data have had past values subtracted), and $q$ is the order of the moving-average model based on the fit statistics in Table 3. In all cases, ARIMA forecasting performed only as well as a random forecast, and ARIMA models were determined not to provide good models of NFT price behavior.

**Table 3:** ARIMA fit statistics for the top 10 ranked NFTs.

Optimized ARIMA parameters	AIC	Sigma-squared	Log-likelihood
ARIMA(4,0,2) with non-zero mean	183,380	1.459e+09	− 91,682
ARIMA(1,1,0)	89,375	97,528,953	− 44,686
ARIMA(1,1,1)	65,715	20,602,420	− 32,855
ARIMA(1,1,0) with drift	60,977	25,360,426	− 30,485
ARIMA(1,1,1)	55,134	31,767,567	− 27,564
ARIMA(3,1,3)	47,117	18,630,823	− 23,551
ARIMA(1,1,0)	35,505	40,573,368	− 17,751
ARIMA(1,0,0) with non-zero mean	30,517	43,399,359	− 15,256
ARIMA(0,0,0) with non-zero mean	28,998	632,708,194	− 14,497
ARIMA(1,1,0) with drift	29,599	35,714,201	− 14,797

### Fractal behavior in the NFT price series

Elliott Wave theory is claimed to provide a fractal-like method for predicting the length and frequency of ‘bull’ and ‘bear’ market swings, perceiving a recursive ‘fractal’ price structure in asset markets^43^. Elliott’s work as been a factor in related studies of asset price time and frequency resolution^71–74^. Until recently, fractal algorithm implementations have been unavailable for analyzing large price datasets such as the one used in this research.

The current research analyzes the multifractal spectrum of Hurst exponents *H* of the price series, a measure of long-term memory of time series, using R’s MFDFA^83^ package. For self-similar time series (e.g., Elliott waves) *H* is directly related to fractal dimension, *D*, where $1 < D < 2$, such that $D = 2 - H$. The values of the Hurst exponent vary between *0* and *1*, with higher values indicating a smoother trend, less volatility, and less roughness^84^. A multifractal system is a generalization of a fractal system in which a single exponent (the fractal dimension) is not enough to describe its dynamics; instead, a continuous spectrum of exponents (the so-called singularity spectrum) is needed^85^. Multifractal systems are common in human behavior and elsewhere in nature. They include the length of coastlines, mountain topography, fully developed turbulence, real-world scenes, heartbeat dynamics, human gait and activity, human brain activity, and natural luminosity time series.

In a multifractal system the behavior around any point is described by a local power law $s\left( {q + a} \right) - s\left( q \right) \sim a^{h\left( q \right)} .$ The exponent $h_{q} = h\left( q \right)$ is called the singularity exponent, as it describes the local degree of singularity or regularity around the point $q$. Multifractals tend to be scale invariant yielding power-law behaviors, self-similarity, and multiscaling^86^. The curves $\left[ {D_{q} ,h_{q} } \right]$ (such as shown in Fig. 8) are called singularity spectra and fully describes the statistical distributions of the price series.

Were the fractal perspective of Elliott Waves to be correct, we would expect each of the top 10 NFTs to have the same singularity spectrum, one that is defined by the Elliott Wave behavior of five upward steps and three downward steps at multiple scales. In fact, each of the singularity spectra in Fig. 8 define significantly different spectra, and thus different behavior in the markets for the 10 most traded NFTs. Fractal analyses of NFT price series do not show consistent, comparable fractal behavior for different NFTs, and thus is not suitable for prediction of future price series based on prior behavior.

### Wavelet decomposition as a method of identifying Elliott Wave and Fractal behavior

Wavelets analyze data in a time–frequency domain, rather than just frequency (as with Fourier analysis), or time (as with ARIMA), and offer a comprehensive mathematical model in that domain. Wavelets hold the potential for extracting specific causal relationships that occur repeatedly over limited time-periods, and which are predictable, repeatable and systemic. Thus they appear to be a very promising candidate for rigorously modeling Elliott waves in price series, which are claimed to reflect investor group psychology. Wavelets are able to fully model patterns, such as those claimed in Elliott wave theory, that appear at multiple scales with neither beginning nor ending. The software and computing power available before a decade ago was inadequate for a thorough study of Elliott waves, which is likely one reason that so few studies have yet taken this approach. Analysis by the many adherents who have invested time and money in understanding Elliott wave patterns has up to this point been implemented using either linear models or using Fibonacci sequences, both computationally less intensive approaches. The current research analyzed wavelets using *R*’s *WaveletComp*^87^ package, which was able to fully capture the salient features of Elliott wave theory. Figure 9 shows the price timeseries for the top 10 NFTs analyzed in this research.

The graph in Fig. 10 analyzes autocycles in the price series of each of the top 10 NFT analyzed in this research. We would expect Elliott Waves to present themselves as autocycles (comparable to autocorrelations) on one of the power spectra, i.e., the color corresponding to the scale at the right at a particular periodicity on the y-axis. The minute-by-minute price series of each of the top 10 NFT was analyzed to compute their power spectra between each possible set of two points on the price series, i.e. their autocycles. The important features of intraday trading are captured in the chosen periodicity time scale which span from 4 min to about 8 h (512 min). Longer weekly cycles were found for all of these NFTs, but these are artifacts that are common for most asset price series. There is very little activity before the end of 2019 in any of these NFTs. Where there were significant autocycles in pricing (the red “high power” funnels) spanning all periods that would appear randomly in time. These reflect times where specific NFTs were being strongly promoted and where there was significant media hype around the particular NFT.

Though there were between three and nine brief periods of high volume, highly autocorrelated activity in each of the NFT price streams, there were no dates that were consistent across all of the price streams. Individual NFTs exhibited their own specific idiosyncratic price autocycles.

The graphs in Fig. 11 show the analysis of general autocyclic power of the Ether, Bitcoin and the whole NFT market. These were small and power spectra are uninformative, supporting a common assertion that prices are statistically independently distributed over time. The next section looks at interactions between cryptocurrency and NFT prices. The current research found that Ether price had little influence on NFT prices, even though NFTs on the OpenSea platform were almost always implemented on the Ethereum blockchain.

I concluded from these analyses that price behavior is strongly tied to the asset and its community of fans/traders. These traders will exhibit periodic idiosyncratic bouts of trading which cools for a while, and then restarts. But wavelet analysis provides no discernible indication of Elliott Wave behavior across the full price series of any of these NFTs.

### Trading interactions between cryptocurrencies and individual NFTs

Prior research^49^ asserted that when Bitcoin and Ether experienced large price and volume movements, NFT prices and trading volume also tended to rise or fall. Additionally, NFT prices were significantly higher when the market sentiment was positive, as measured by the *Crypto Fear and Greed Index*. NFT traders displayed hedging behavior, as they tended to sell NFTs and move into Bitcoin or Ether during periods of high volatility or uncertainty in the cryptocurrency markets, suggesting that NFT traders might use Bitcoin and Ether as a hedge against market risk. If hedging^49^ into and out of Ether and Bitcoin exists in NFT markets, then they should show up in wavelet analysis. Wavelets offer an alternative to the direct causality of econometrics, by additionally providing lead and lag information embedded in the phase difference between the two variables. Wavelets analyze time in terms of coherency, start, duration and phase. Phase can be though of in terms of causality plus a time (lead-lag) component. No cause and effect relation is instantaneous—even physical systems’ cause effect is limited to the speed of light (e.g., gravitational attraction). Phase adds the time component to causality, and essentially extends empirical analysis of causality by adding temporal information.

**Figure 14:**
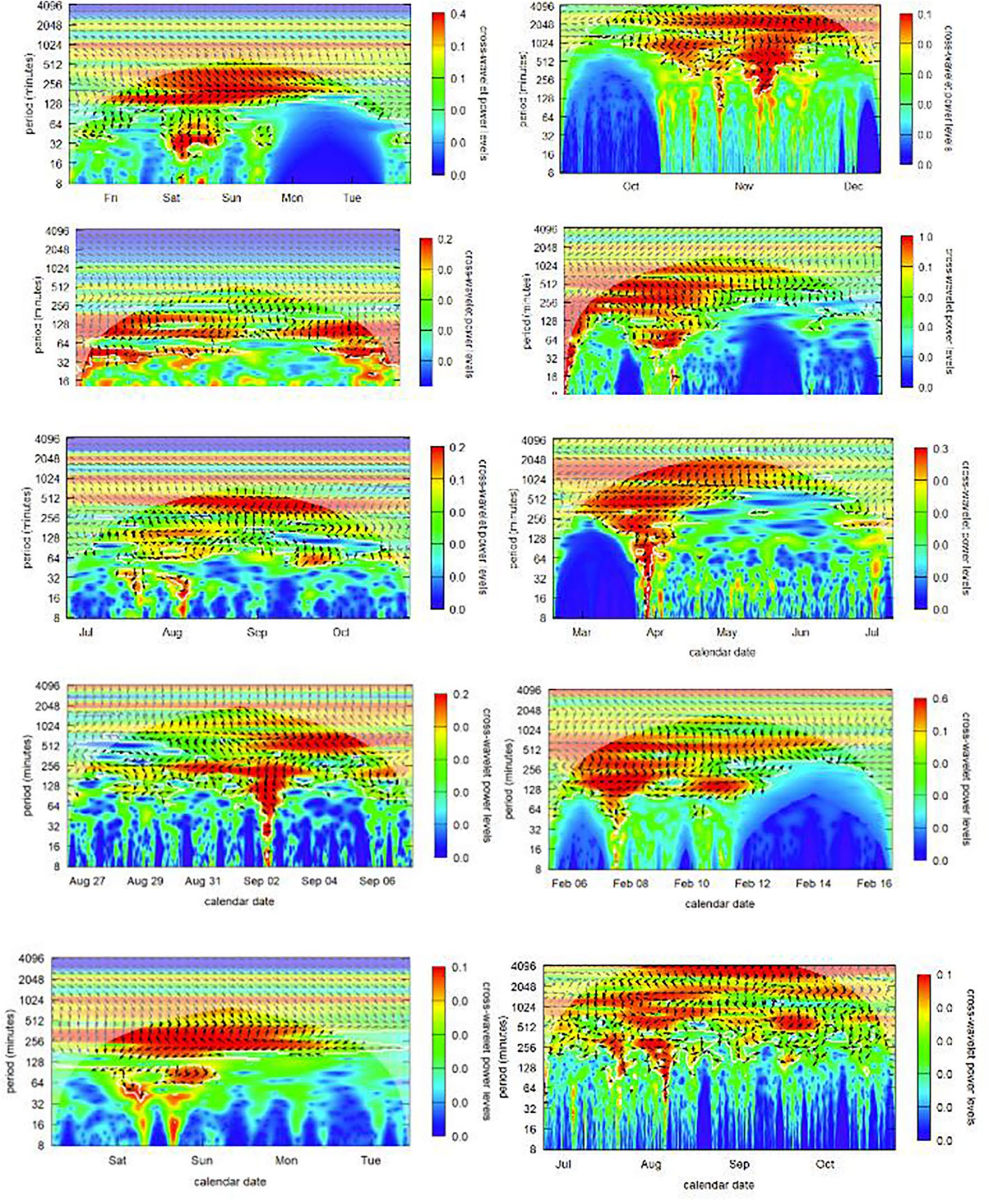
Bitcoin price coherency (correlation) with the Top 10 ranked ranked NFTs.

The graphs in Figs. 12 and 14 analyze these cryptocurrency-NFT price series couplings for of each of the top 10 NFT analyzed in this research. They generally support prior findings for price cycles of more than one trading day (i.e., 8 h) and provide detail on the nature of hedging behavior. Ante’s^49^ suggests that such coupling would be significant, and though this seems to be borne out at longer periodicities, it is nonexistent for periods shorter than a day (i.e., 8.5 h or 512 min). A useful analogy for understanding wavelet coherency is music’s “call and response.” In call and response, a group chorus (e.g., the market collectively) sings a motif, possibly with chordal accompaniment, and a few beats later, a soloist (e.g., an investor/trader) responds in answer. Call and response, occurs over multiple pitches (frequencies or periodicities) over multiple beats (varying time periods). Each of the powers of 2 represents an ‘octave’ and a resolution of $\frac{1}{12}$ was used on the y-axis. Wavelet *coherency* of a time series in is analogous to the coefficient of correlation $\rho$ in linear statistical models. That “call and response” appears at long intervals in Figs. 12 and 14, but not in intraday trading.

The next two sections look at NFT-cryptocurrency coherencies for Bitcoin and Ether. Because most NFTs are traded on the Ethereum blockchain, and most trades are in Ether cryptocurrency, I investigated cycles in Ether cryptocurrency, and later, coherence between Ether cycles and the top three “art” NFTs. Post-Everydays, Ether autocycles appear around 2048, 1024 and 256 min cycles (around 32 h, 17 h and 4 h). In contrast, Bitcoin dominates cryptocurrency markets and is somewhat of a de facto numeraire commodity, and thus the same analysis was applied to Bitcoin (Fig. 14). Across its full range, cycles appear around 2048 and 1024 min cycles (1.5 days and 16 h).

#### Ether

NFT-Ether coherency is graphed in Fig. 12. Note that in Fig. 12 and also in Fig. 14, the x-axis time values show various ranges and are not consistent. These graphs do not all cover the same time period, but for the NFT-cryptocurrency coherency analysis this was not important, as only the periodicity of the NFT-cryptocurrency relationship is being elicited. Figure 12’s cross-wavelet coherency power spectra generally supports Ante’s^49^ conclusions, but provides significantly more detail on how these take place in practice. Ethereum was the first blockchain to support smart contracts, and because of that, the first to support NFTs. Almost all NFTs are traded on the Ethereum blockchain, thus analysis was restricted to the Ethereum blockchain.

Inspection of coherency power spectra between Ether and the top 10 NFTs reveals common patterns in trading:

- The black arrows on the diagram indicate the phase $\omega$ of the coherency with trading from Ether into the NFT (generally right-pointing arrows) and out of the NFT (generally left-pointing arrows) with the degree of rotation indicating lead-lag times dependent on the period (y-axis)
- At periods greater than 48 h (2880 min) coherency is low, implying that trade decisions are generally made within a daily time frame.
- At periods greater than 24 h (1440 min) and less than 48 h there is significant into and out of Ether and that specific NFT, with a latency (the degree of rotation of the phase arrows on the chart) that evolves over time. One explanation of this evolving latency is that since specific NFTs are popular during a relatively limited period of time where they are heavily traded, that these phase rotations reflect the transfer of an Ether account holding into the NFT and then later out of the NFT holdings. Additionally since NFT price increases with popularity, more Ether needs to be traded for each transaction. All of the 10 charts show almost a complete phase rotation over their trading life, reflecting the trade into, holding and trade out of that NFT.
- OpenSea explains on their website that the average time it takes to process a transaction is around an hour. Additional time may be required for the ‘gas’ fees (transaction processing fees on the Ethereum blockchain) which are associated with an OpenSea transaction. Thus it is difficult for Ether-NFT portfolio balancing to take place in less that two hours.
- NFTs experienced periods of frenzied trading (the red funnels in the charts) between one and about six times in their trading existence. Trading activity into and out of Ether occurred within 2 h (~ 128 min) and 8 h (~ 512 min) period cycles. Phase arrows show a trading from Ether into the NFT (rightward pointing arrows) and out of the NFT (rightward pointing arrows).
- Trading platforms can be seen as a form of social media, with faddish trends directly impacted in prices. NFT trading behavior clearly indicates their faddish nature. NFT fads arise suddenly and arbitrarily, are quick-spreading and short-lived, quickly reversing themselves. In other social media, general novelty, mass marketing, peer pressure, and the desire to trendy drive interest and transactions. Furthermore, the NFT market is very thinly traded (see Figs. 1 and 2 on price and volume) with 99% of NFTs being traded less than 5 times. NFT markets like OpenSea have much more in common with social media platforms like TikTok, rather than with a liquid asset market like the New York Stock Exchange. TikTok is a short-form video hosting service owned by ByteDance. It hosts user-submitted videos, which can range in duration from 3 s to 10 min. Cloudflare ranked TikTok the World’s most popular website of 2021, surpassing Google.

#### Bitcoin

NFT-Bitcoin coherency is graphed in Fig. 14. Note that in Fig. 14, the x-axis time values show various ranges and are not consistent. These graphs do not all cover the same time period, but for the NFT-cryptocurrency coherency analysis this was not important, as only the periodicity of the NFT-cryptocurrency relationship is being elicited. Figure 14 presents the cross-wavelet coherency power spectra, revealing that observations made for the Ether-NFT coherency and trading patterns generally apply to Bitcoin-NFT coherency. Linkages between two markets are causal, in the mutual information sense of causality, and the direction of the causality is determined by whether phase analysis indicates a lead or lag relationship (the causal arrow has its arrowhead pointed to the lagging variable). The time-lag associated with the causal relationship is determined by the degree that the cycles are out of phase.

- In a comparison of Ether-NFT coherency to Bitcoin-NFT coherency, we see trading (red funnels) taking place at roughly the same time and periodicity in and out of either Bitcoin or Ether, and at roughly the same cross-wavelet power levels (color legend on the right hand side).
- Bitcoin trades seem to be taking place a bit faster (~ 1 h faster) than Ether trades, suggesting that computing ‘gas’ on the Ethereum blockchain may impose a time lag as suggested earlier.

#### Phase shifts

**Figure 15:**
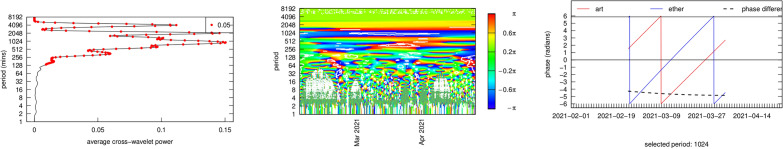
Price coherency (correlation) with ether for the 1st ranked NFT.

**Figure 16:**
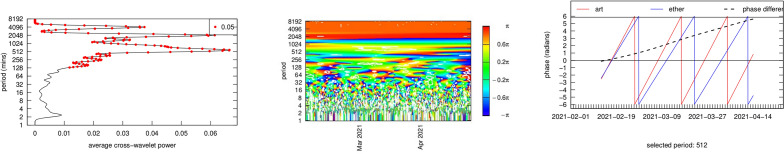
Price coherency (correlation) with ether for the 2nd ranked NFT.

Figures 15 and 16 provide phase and power profiles for the two most heavily traded NFTs in the dataset, where I had sufficient data to compute these graphs. These graphs provide a more detailed explanation of the small directional arrows showing causal direction on the graphs in Figs. 12 and 14. “Phase” measures the relative time difference between two sinusoidal timeseries functions. The phase lag is one component of these functions and occurs when a system response lags behind a signal. The phase $\omega$ information contained in the black arrows on analyses of the top 10 NFTs provides information about trading lead-lag times and direction. This is somewhat complicated to read from the charts since the period (y-axis) determines the exact timing. For example at a periodicity of 256 mins, a phase of $\omega = \frac{\pi }{4}$ gives $lag = \frac{\omega }{2\pi } \times period$ or 32 min. Since this information tends to be very time specific—it is not invariant across the entire period of trading in a specific NFT—it was felt that investigating the top two NFTs would be sufficient to review whatever additional information might be gleaned from detailed phase analysis. These figures display phase information for the Ether price stream and the first and second most traded NFTs. Each of these two figures shows the power curve (the red dots indicate which parts are statistically significant at the 5% level) a phase diagram (colors indicate the lead or lag in time in on a scale of $\left[ { - \pi ,\pi } \right]$ minutes) and a phase lag chart for the period of most active trading for each particular NFT.

$Ether - NFT_{rank1}$ analysis shows trading in ~ 1024 min periods where NFT price lags Ether price by about 8 radians $\approx$ 1400 min (~ 1 day). Otherwise, there appear to be no linked price cycles in the two asset classes. $Ether - NFT_{rank1}$ analysis shows trading in ~ 16, 128 and 512 min periods during the period January through August of 2020, during what was called the “crypto winter.” Period 16 showed showed almost no NFT price lags. Period 128 showed NFT price lags Ether price by about 5 radians in January 2020, or $\approx$ 1 h. Period 512 showed NFT price lags Market price by about 5 radians in September 2020, or $\approx$ 2 h, but by only around 10 min in early 2020. This, again, was a time of intense portfolio reassessment in the Bitcoin market, and that may have had an effect.

$Ether - NFT_{rank2}$ analysis shows trading in ~ 512 min periods where NFT price leads Ether price by a rising trend of about 0 radians from 2-19-2021 through about 6 radians on 4-14-2021 (6 radians $\approx$ 512 min (~ 8 h). Otherwise, there appear to be no linked price cycles in the two asset classes.

#### Interactions between the whole NFT market and individual NFTs

There is a tradition in asset market research of analyzing the influence of overall market sentiment on individual asset prices. The Capital Asset Pricing Model^48^ (CAPM) represents the most well-know of these approaches, and is used in separating risk into market and firm-specific risk (i.e., the so-called $\alpha$ and $\beta$). In the current context I looked at the influence of the composite price movements of all NFT assets, versus individual price movements in the top traded three NFTs, and call these the NFT whole market effect.

**Figure 17:**
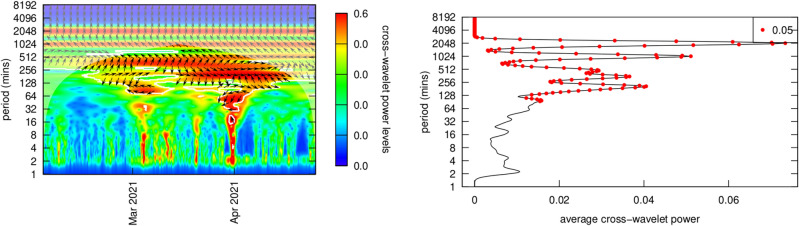
Price coherency (correlation) of the 1st rank NFT with the whole NFT market.

**Figure 18:**
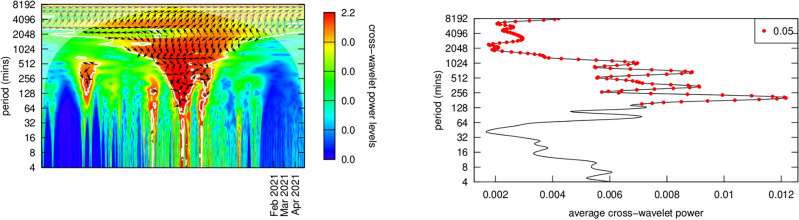
Price coherency (correlation) of the 2nd rank NFT with the whole NFT market.

**Figure 19:**
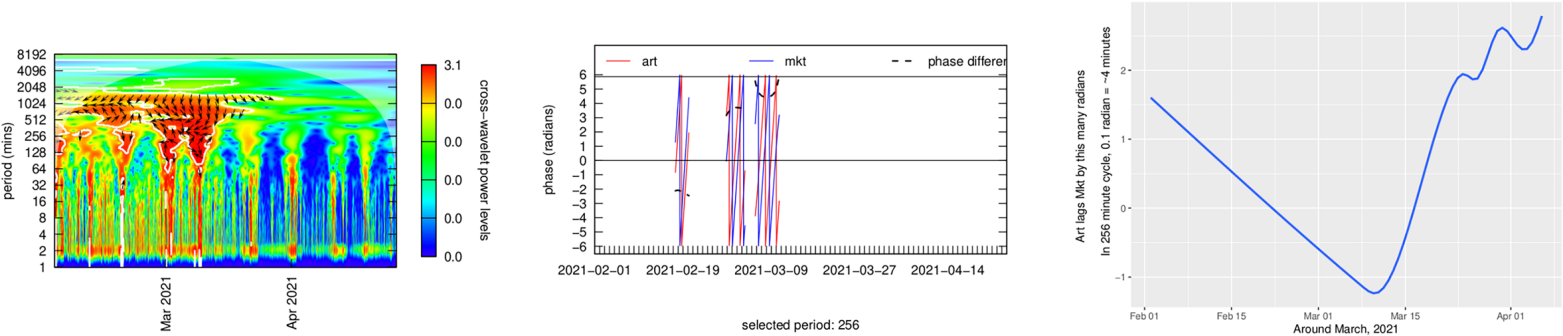
Price phase lags with Whole NFT market of 2nd ranked NFT.

Figures 17 and 18 analyzed price coherency between the most traded and second most traded NFT and the whole NFT market. Figure 19 provides power and coherency statistics for the influence of Whole NFT market influences on the second most traded NFT (the most traded NFT showed no phase lags at all, and thus the graph was noninformative for coherency). In looking at these, I have restricted the power spectrum graph to time periods in which there was active trading, and have placed arrows on the graph indicating the phase difference of the NFT vs. Market price series on a scale of $\left[ { - \pi ,\pi } \right]$. At the far right of the figures is the power curve showing significant coherency power cycles. Figure 19 investigates high volume trading in one part of the second top NFT coherency power spectrum graph. The middle graph converts the arrows to a phase lag-lead on a scale of $\left[ { - \pi ,\pi } \right]$ showing how the NFT and the market diverge. This divergence is isolated on the graph at the far right which shows the evolution of the lag-lead times for that particular short-term cycle.

This last bit of analysis is not generalizable, rather it represents detailed analysis of particular cases (a case study) of a single episode of very active trading. What it does reveal is a steady evolution of the informational lead to lag over about of frenzied trading. One can speculate that this in turn represents the diffusion of information, perhaps through forums and bulletin boards, of information and marketing for a specific NFT at a specific time. This is a rich potential area of investigation for future study.

## Findings and conclusions

This research studied the existence of Elliott wave like behavior in NFT markets, using wavelet and fractal models to represent Elliott wave models of technical price movements in OpenSea’s NFT market. The research investigated linear timeseries models as a benchmark for subsequent study of Elliott wave like price autocycles, as well as price cycle correlations between NFTs and cryptocurrencies which have been suggested in the conclusions of prior studies.

There were nine key findings from this research.

1. Linear $ARIMA\left(p,d,q\right)$ time series models do not accurately predict price behavior in NFT markets.
2. Traditional linear finance models, e.g., the capital asset pricing model^48^ (CAPM), do not accurately predict price behavior in NFT markets.
3. Fractal analyses of NFT price series do not show consistent, comparable fractal behavior for different NFTs, and thus holds little potential for prediction of future NFT price series.
4. Traders do exhibit periodic idiosyncratic bouts of trading; this abates, and then restarts. But there is no indication of Elliott Wave behavior across the full price series of individual NFTs.
5. Ante’s^49^ conclusions that Bitcoin and Ether cryptocurrency market movements and NFT price and volume were supported. I did not test other cryptocurrencies.
6. The influence of cryptocurrency prices and trading was low to nonexistent for periods longer than 48 h.
7. At periods between 24 and 48 h there is significant influence of cryptocurrency prices and trading into and out of Ether from and to specific specific NFTs, with a latency that evolves over time. These effects may reflect social media and news activities which was not available in the research.
8. There is an increase in trades into and out of NFTs every two and eight hours. These may reflect processing times and regulations on OpenSea, but we were not able to verify that assertion.
9. NFT price generally increased with trading volume.

The following conclusions were drawn from this research.

ARIMA provided a benchmark linear model for predicting prices. I applied to each individual NFT dataset an optimal $ARIMA\left( {p,d,q} \right)$ model, where $p$ is the order (number of time lags) of the autoregressive model, $d$ is the degree of differencing (the number of times the data have had past values subtracted), and $q$ is the order. In all cases, ARIMA forecasting performed only as well as a random forecast, and ARIMA models were determined not to provide good models of NFT price behavior. *I conclude that linear models are not useful in modelling NFT price series.*

For the fractal modeling approach to validating Elliott Wave behavior to be correct, we would expect each of the top 10 NFTs to have the same singularity spectrum, one that is defined by the Elliott Wave behavior of five upward steps and three downward steps at multiple scales. In fact, each of the singularity spectra exhibit dramatically different behavior over the 10 most traded NFTs. Fractal analyses of NFT price series do not show consistent, comparable fractal behavior for different NFTs, and thus holds little potential for prediction of future price series based on prior behavior. *I concluded that Elliott Wave behavior modeled as a fractal is not useful in predicting NFT price series.*

Elliott Wave behavior may also be modeled using wavelet autocycles. My investigation of autocycles in the Ether, Bitcoin and NFT markets showed that these were not significant factors in the market. Except for showing longer term daily and weekly cycles, these autocycle power spectrum charts are just busy messes involving small periods of active trading. The NFT market as a whole had a power spectrum that was mostly empty and the market only sees flashes of activity. Compared to the autocycles in the individual NFTs, the maximum power of an autocycle on any of these three charts is small—one to two orders of magnitude smaller than for individual NFTs. *My investigation of autocycles does not support the existence of Elliott Wave behavior in NFT markets. I also concluded from this that price behavior is strongly tied to the asset and its community of fans/traders. These traders will exhibit periodic idiosyncratic bouts of trading which cools for a while, and then restarts. But there is no indication of Elliott Wave behavior across the full price series of individual NFTs.*

There is a tradition in asset market research of analyzing the influence of overall market sentiment on individual asset prices. The Capital Asset Pricing Model represents the most well-know of these approaches, and is used in separating risk into market and firm-specific risk ($\alpha$ and $\beta$). In the current context I looked at the influence of the composite price movements of all NFT assets, versus individual price movements in the top traded three NFTs, calling these the NFT whole market effect. *There is no evidence supporting CAPM-type market effects across the full price series of individual NFTs.*

Ante’s^49^ research concluded that traders were balancing their portfolios by trading into and out of NFT and cryptocurrencies (mainly Ether, since most NFTs are on the Ethereum blockchain). I investigated this at length using a variety of methodologies from wavelets analysis. The Ether-NFT for individual NFTs was investigated for the top 10 NFTs. At periods greater than 48 h (2880 min) coherency is low, implying that trade decisions are generally made within a daily time frame. At periods greater than 24 h (1440 min) and less than 48 h there is significant trading into and out of Ether and that specific NFT, with a latency (the degree of rotation of the phase arrows on the chart) that evolves over time. One explanation of this evolving latency is that since specific NFTs are popular during a relatively limited period of time where they are heavily traded, that these phase rotations reflect the transfer of an Ether account holding into the NFT and then later out of the NFT holdings. Additionally since NFT price increases with popularity, more Ether needs to be traded for each transaction. All of the 10 charts show almost a complete phase rotation over their trading life, reflecting the trade into, holding and trade out of that NFT. NFTs experienced periods of frenzied trading (the red funnels in the charts) between one and about six times in their trading existence. Trading activity into and out of Ether occurred within 2 h (~ 128 min) and 8 h (~ 512 min) period cycles. Phase arrows show a trading from Ether into the NFT (rightward pointing arrows) and out of the NFT (rightward pointing arrows). OpenSea’s two hour lower limit on effects is probably the result of OpenSea having an average time to process a transaction is around two hours, counting issues with ‘gas’ that make it difficult for Ether-NFT portfolio balancing to take place in less that two hours. In a comparison of Ether-NFT coherency to Bitcoin-NFT coherency, we see trading (red funnels) taking place at roughly the same time and periodicity in and out of either Bitcoin or Ether, and at roughly the same cross-wavelet power levels (color legend on the right hand side). Bitcoin trades seem to be taking place a bit faster (~ 1 h faster) than Ether trades, suggesting that computing ‘gas’ on the Ethereum blockchain may impose a time lag as suggested earlier. Activities in the NFT market appear in periods of around 2, 4, 8, 16 and 32 h; and additionally a general 7 day cycle. These represent periods where the activity in another market is coupled (coherence) to the NFT market at a statistically significant level. *My analysis of trading coherency strongly supports Ante’s*^49^* conclusions on cryptocurrency market movements and NFT price and volume. NFT prices and trading volume are influenced over time periods greater than a day by the price and volume movements of Bitcoin and Ether.*

## Future research

Trading platforms can be considered as a form of social media, as they enable users to interact and share information with each other, which can directly impact prices. NFTs, in particular, have exhibited faddish behavior, with sudden and arbitrary trends emerging and spreading quickly through social media channels. These trends are often short-lived, and prices can reverse themselves just as quickly. The factors that drive interest and transactions in NFTs are similar to those found in other social media platforms like TikTok, such as novelty, mass marketing, peer pressure, and the desire to be trendy. As a result, NFT markets like OpenSea are much more akin to social media platforms like TikTok than to liquid asset markets like the New York Stock Exchange.

Price movements in a market reflect the dissemination of information among traders, who may or may not act on that information. The effects we see in this research, the leads and lags in pricing, the cycles, autocycles and influences, are indicators of the way that information moves among traders. Autocycles suggest that traders are looking at prior price streams through the lens of some model that will yield forecasts. Coherency in NFT-cryptocurrency cycles suggests that traders are drawing information from other markets, or are balancing portfolios.

It is worth noting that the NFT market is still relatively new and thinly traded, with all but a handful of NFTs being traded less than five times. This lack of liquidity can make it more difficult to predict price movements and can increase the potential for volatility. However, if the market matures and more participants enter, it is possible that liquidity may improve and trading patterns may become more predictable.

The current research paper applies wavelet and fractal analysis to the largest dataset of NFT data from the OpenSea market yet studied in a research paper. This research adapted existing models of group trading behavior that are used in industry, and restated them in terms of mathematically rigorous wavelet and fractal analysis. These methodologies are able to extract considerably more information, and can set up statistical tests that were not conducted in Ante’s^49^ and Nadini et al.’s^82^ research, which provided the context for this research project. Ante^49^ and Nadini et al.^82^ relied heavily on descriptive statistics and graphs, as well as linear models to explore a subset of the current OpenSea data, and were to some extent model-agnostic. The current research is specifically investigating the validity of an Elliott wave model for NFT markets.

The current research showed the validity of Elliott wave price cycles in the NFT market, but fell short of offering accurate prediction models. Future research is needed to more effectively predict market movements, both for individual NFTs as well as for market segments and the market as a whole. Such models are need for government regulation and for the establishment of successful investment funds.

Prediction models are also important at an individual trader level because they allow traders to fully inject all available price information into the market’s price series. Liquidity providers and speculators in particular are essential for maintaining fair pricing and volume of trades in a market. Currently NFT markets are highly illiquid, where assets may be over or underpriced, without every reaching an equilibrium price that can attract buyers and sellers and clear the market. The health and profitability of NFT markets in the future requires a extension of the current research to develop prediction models that can inject all the available information into the market prices.

## Data Availability

All of the datasets that I have used in this paper are available for download at my GitHub site:https://github.com/westland/nft_periodicity. If you have any questions concerning the datasets, please forward these questions to me.
